# Risk Factors for Post-ERCP Pancreatitis: Impact of Transpancreatic Septotomy, Needle–Knife Precut, and Duodenal Diverticulum in 1226 Procedures

**DOI:** 10.3390/jcm15020504

**Published:** 2026-01-08

**Authors:** Mehmet Kasım Aydın, Mehmet Cudi Tuncer

**Affiliations:** 1 Department of Gastroenterology, Faculty of Medicine, Mersin University, Mersin 33110, Turkey; 2 Department of Anatomy, Faculty of Medicine, Dicle University, Diyarbakir 21280, Turkey; drcudi@hotmail.com

**Keywords:** endoscopic retrograde cholangiopancreatography, post-ERCP pancreatitis, transpancreatic septotomy, needle–knife precut, ERCP complications

## Abstract

**Background:** Post-ERCP pancreatitis (PEP) remains the most common and clinically relevant adverse event following endoscopic retrograde cholangiopancreatography (ERCP). The impact of periampullary duodenal diverticulum and advanced cannulation techniques—particularly needle–knife precut sphincterotomy and transpancreatic septotomy (TPS)—on PEP risk remains debated. This study aimed to evaluate the association of these factors with PEP development in a large tertiary-center cohort. **Methods:** This retrospective study included 1226 patients who underwent ERCP between January 2018 and October 2022. Demographic, clinical, and procedural variables were recorded. Outcomes included PEP, hyperamylasemia, bleeding, and perforation. Univariable analyses were followed by multivariable logistic regression to identify independent predictors of PEP. Adjusted odds ratios (aORs) with 95% confidence intervals (CIs) were calculated. **Results:** PEP occurred in 17.3% of the cohort. Needle–knife precut sphincterotomy and transpancreatic septotomy were frequently used advanced cannulation techniques and were both associated with an increased prevalence of PEP, with PEP occurring in 30.3% of patients undergoing needle–knife precut sphincterotomy and 56.9% of those undergoing transpancreatic septotomy. In the multivariable model, needle–knife precut independently increased PEP risk by 2.45-fold (aOR 2.45; 95% CI 1.78–3.36; *p* < 0.001), whereas TPS demonstrated the strongest association, increasing the risk nearly fivefold (aOR 4.92; 95% CI 2.98–8.11; *p* < 0.001). Female sex showed a nonsignificant trend toward increased PEP risk (aOR 1.28; 95% CI 0.96–1.69; *p* = 0.08). Periampullary duodenal diverticulum, pancreatic duct stenting, comorbidities, and age were not independently associated with PEP development (*p* > 0.05 for all). **Conclusions:** Needle–knife precut sphincterotomy and transpancreatic septotomy were independent predictors of PEP, with the highest risk observed for transpancreatic septotomy, whereas periampullary diverticulum and pancreatic duct stenting were not associated with increased risk.

## 1. Introduction

ERCP is a widely used interventional procedure for both the diagnosis and treatment of biliary and pancreatic disorders. However, serious complications such as PEP, bleeding, perforation, and cholangitis may occur, significantly increasing patient morbidity and mortality [1,2]. Therefore, identifying clinical and procedural risk factors that may predict ERCP-related complications is of critical importance for optimizing patient safety and procedural outcomes.

The presence of a duodenal diverticulum, particularly when located periampullary, may challenge papillary visualization and cannulation, potentially prolonging the procedure and increasing the risk of adverse events [3,4]. Nonetheless, other investigations have found no significant association between diverticula and ERCP-related complications, leaving the clinical relevance of this anatomical variant a subject of ongoing debate [5].

Advanced cannulation techniques, especially needle–knife precut sphincterotomy and TPS, are frequently employed in cases of difficult cannulation and may themselves influence complication risk. The TPS technique, first described by Goff, involves controlled pancreatic duct access to facilitate biliary entry and has been suggested as an effective alternative in experienced hands [6,7]. Placement of a prophylactic pancreatic stent has been shown in several meta-analyses to reduce the risk of PEP [8]. However, because stents are typically placed in patients at inherently higher risk for PEP—including those undergoing difficult cannulation or advanced access techniques—their protective effect may not always be evident in real-world clinical settings, as demonstrated in a recent individual patient data meta-analysis showing no significant risk reduction after adjustment for baseline patient characteristics [9].

Individual patient characteristics, including age, sex, and chronic comorbidities, may also influence complication susceptibility; however, the magnitude of their contribution remains uncertain across heterogeneous clinical cohorts, as demonstrated in large prospective and multicenter studies [10]. Given these limitations in the existing literature, further clarification of risk factors related to techniques such as precut sphincterotomy, transpancreatic septotomy, periampullary diverticulum, and pancreatic duct stenting remains necessary.

Recent high-quality evidence offers important contemporary insights into the multifactorial determinants of PEP. A comprehensive meta-analysis by Ding et al. demonstrated that both patient-related and procedure-related variables—such as female sex, previous pancreatitis, difficult cannulation, precut sphincterotomy, endoscopic sphincterotomy, and main pancreatic duct injection—significantly increase PEP risk [11]. More recently, an extensive systematic review and adjusted-effects meta-analysis including over 300,000 ERCP procedures confirmed difficult cannulation, pancreatic duct manipulation, and precut sphincterotomy as strong independent predictors of PEP, providing more precise pooled estimates of aORs [12].

In parallel, new data evaluating TPS across different clinical practice environments indicate considerable variability in outcomes: studies from both low- and high-volume centers report improvements in cannulation success with TPS, though variation in operator experience and patient selection continues to influence its safety profile [13,14].

Importantly, an individual patient data meta-analysis has recently challenged the traditionally held assumption that prophylactic pancreatic stenting reliably reduces PEP risk; after adjustment for patient-level characteristics, pancreatic duct stents demonstrated no significant benefit, whereas rectal nonsteroidal anti-inflammatory drugs (NSAIDs) and aggressive intravenous hydration showed clear prophylactic efficacy [9]. Together, these contemporary findings highlight substantial heterogeneity in real-world ERCP practice and underscore the ongoing need for large, detailed clinical analyses to better clarify how anatomical factors, advanced cannulation techniques, comorbidities, and prophylactic strategies interact to influence PEP risk.

The aim of this study was to investigate the association of duodenal diverticulum, precut and TPS techniques, pancreatic stent placement, and the presence of comorbidities with post-ERCP complications in a large single-center retrospective cohort.

## 2. Methods

### 2.1. Study Design and Ethical Approval

This observational, retrospective study included patients who underwent ERCP at Mersin University Faculty of Medicine Hospital between January 2018 and October 2022. All procedures were performed in accordance with the Declaration of Helsinki, and the study protocol was approved by the Mersin University Clinical Research Ethics Committee (Approval No: 374, 9 April 2025). Because the study utilized anonymized retrospective data, the requirement for informed consent was waived by the committee.

### 2.2. Study Population and Exclusion Criteria

All patients aged ≥18 years who underwent diagnostic or therapeutic ERCP and had complete clinical, laboratory, radiological, and procedural documentation were eligible. Patients were excluded if they were younger than 18 years, had a known pancreatic head mass prior to ERCP, were pregnant, presented with active pancreatitis before the procedure, had a documented severe hypersensitivity to aspirin or NSAIDs, or had a history of gastrointestinal bleeding. After applying these criteria, 1226 patients were included in the final analysis.

### 2.3. Study Flow and Patient Selection Process

**Figure 1:**
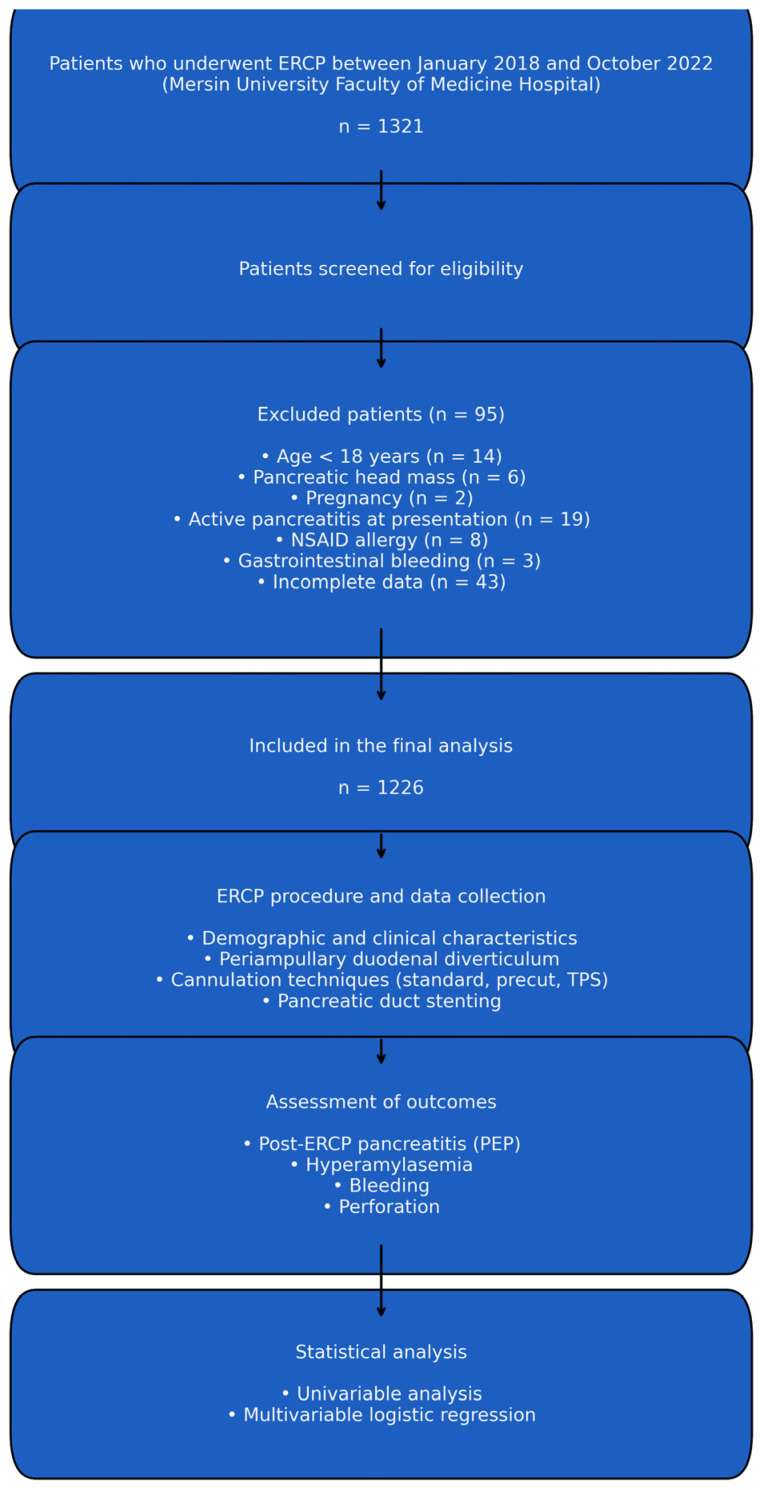
Flowchart illustrating the selection of the study population. A total of 1321 ERCP procedures performed between January 2018 and October 2022 were screened. After excluding patients who were <18 years old (n = 14), had a pancreatic head mass (n = 6), pregnancy (n = 2), active pancreatitis (n = 19), NSAID allergy (n = 8), GIS bleeding (n = 3), or incomplete data (n = 43), a final cohort of 1226 patients was included in the analysis.

The flow of patient selection is summarized in Figure 1. A total of 1321 ERCP procedures performed between January 2018 and October 2022 were screened for eligibility. Patients were excluded according to predefined criteria, including age <18 years, presence of a pancreatic head mass, pregnancy, active pancreatitis at presentation, documented NSAID hypersensitivity, active gastrointestinal bleeding, or incomplete procedural or clinical data. After excluding 95 patients based on these criteria, a final cohort of 1226 patients with complete demographic, clinical, laboratory, imaging, and procedural information was included in the analysis. This stepwise selection process ensured a homogeneous study population and minimized potential sources of misclassification bias.

### 2.4. Data Sources and Variables

Demographic characteristics (age, sex), comorbidities, medication use, history of pancreatobiliary disease, laboratory parameters (amylase, lipase, hemoglobin), imaging findings, and detailed endoscopic procedure reports were obtained from the hospital information system and patient files. All peri-procedural data were recorded prospectively on standardized forms and subsequently verified. Information regarding the systematic use of established prophylactic measures for post-ERCP pancreatitis, including rectal nonsteroidal anti-inflammatory drugs and aggressive intravenous hydration, was not uniformly available in the retrospective dataset and therefore could not be reliably analyzed.

The presence of a duodenal diverticulum was documented endoscopically. Indications for TPS (Goff technique) and needle–knife precut sphincterotomy included difficult papillary access, failed biliary cannulation, or anatomically challenging papillae. Pancreatic duct stent placement was reserved for patients with multiple unintended pancreatic duct cannulations, papillary trauma, or those considered at high risk for pancreatitis.

### 2.5. Definitions and Outcome Measures

The primary outcome was PEP, defined according to Cotton et al. and American/European Society of Gastrointestinal Endoscopy (ASGE/ESGE) guidelines as characteristic abdominal pain occurring within 24 h after ERCP accompanied by serum amylase levels ≥3 times the upper limit of normal [1,15].

PEP severity was categorized using standard criteria [16]:

- **Mild:** hospitalization for 2–3 days
- **Moderate:** hospitalization for 4–10 days
- **Severe:** hospitalization for >10 days or requiring intensive care, surgical, or radiologic intervention

Secondary outcomes included:

- **Hyperamylasemia:** elevated serum amylase without clinical PEP
- **Bleeding:** hemoglobin decrease ≥2 g/dL and/or need for endoscopic hemostasis
- **Perforation:** presence of free or retroperitoneal air on imaging

### 2.6. Endoscopic Advanced Cannulation Techniques

**Figure 2:**
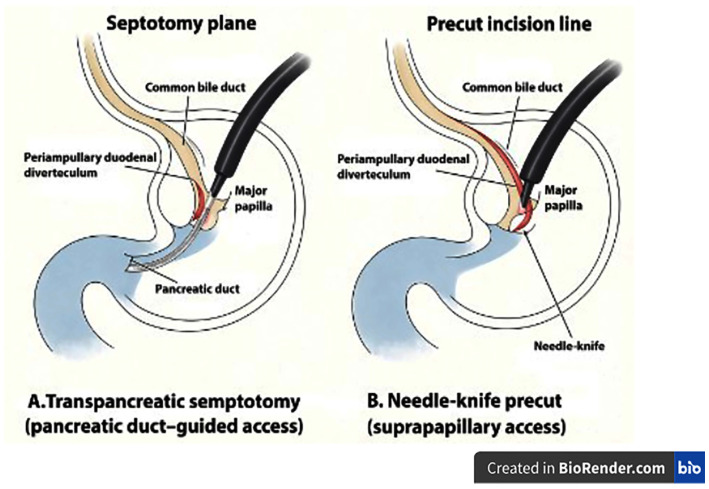
Schematic anatomical illustration of advanced biliary cannulation techniques used during ERCP. (**A**) TPS, in which biliary access is achieved by controlled incision of the pancreaticobiliary septum following intentional pancreatic duct cannulation, including cases with periampullary duodenal diverticulum. (**B**) Needle–knife precut sphincterotomy, in which biliary access is obtained by a suprapapillary mucosal incision directed toward the common bile duct. This simplified schematic illustration is intended to highlight the conceptual differences between TPS and needle–knife precut sphincterotomy and does not aim to represent the full anatomical variability or technical complexity encountered in clinical practice. The figure is original and was created by the authors using BioRender.com.

In cases of difficult biliary cannulation, two advanced cannulation techniques, TPS and needle–knife precut sphincterotomy, were employed at the discretion of the attending endoscopist based on procedural difficulty, anatomical considerations, and real-time intra-procedural assessment (Figure 2). In routine clinical practice, difficult biliary cannulation was defined as failure to achieve selective biliary access using standard cannulation techniques, leading to the need for escalation to advanced access methods. Objective parameters such as cannulation time, number of cannulation attempts, or frequency of unintended pancreatic duct cannulations were not consistently available due to the retrospective nature of the study and therefore could not be systematically analyzed. No predefined or uniform thresholds for escalation to advanced cannulation techniques were applied; rather, the decision to perform precut sphincterotomy or transpancreatic septotomy was individualized and based on real-time procedural judgment. TPS was performed after intentional cannulation of the pancreatic duct, followed by a controlled incision along the pancreatic–biliary septum to expose the axis of the common bile duct and facilitate biliary access. In contrast, needle–knife precut sphincterotomy involved creating an external mucosal incision over the papillary roof toward the direction of the common bile duct to achieve selective biliary entry when standard cannulation techniques were unsuccessful. Pancreatic duct stenting was considered in patients with repeated unintended pancreatic duct cannulations or papillary trauma; however, detailed TPS-specific data regarding stent use frequency and technical characteristics were not consistently available in the retrospective dataset. The presence and orientation of a periampullary duodenal diverticulum were documented during the procedure, as this anatomical variation may influence papillary position and contribute to cannulation difficulty.

### 2.7. Confounding Variables

Potential confounders included age, sex, comorbidities, indication for ERCP, medication use, and history of pancreatobiliary disease. All ERCP procedures were performed by experienced endoscopists working in a tertiary referral center. Operator-related variability was also recognized as a potential confounding factor due to differences in endoscopist experience. However, endoscopist-level variables such as individual procedural volume and years of experience were not available for quantitative analysis and were therefore not included in the multivariable models.

### 2.8. Statistical Analysis

Statistical analyses were performed using IBM SPSS Statistics version 21.0 (IBM Corp., Armonk, NY, USA) and MedCalc Statistical Software version 23.4.5 (MedCalc Software Ltd., Ostend, Belgium). Continuous variables were assessed for normality and are presented as mean ± standard deviation (SD) or median (minimum–maximum), as appropriate. Categorical variables are expressed as frequencies and percentages. Associations between categorical variables were evaluated using Pearson’s chi-square test. Fisher’s exact test or Yates’ continuity correction was applied when the assumptions of the chi-square test were not met. A two-sided *p* value < 0.05 was considered statistically significant.

To identify independent predictors of PEP, a multivariable logistic regression model was constructed. Potential confounding variables were identified a priori based on clinical relevance and previously published literature. Variables entered into the multivariable model included age, sex, presence of duodenal diverticulum, needle–knife precut sphincterotomy, TPS, pancreatic duct stenting, and chronic comorbidities. aORs with corresponding 95% CIs were calculated using Wald statistics. Model adequacy was assessed using the Hosmer–Lemeshow goodness-of-fit test and the Nagelkerke pseudo-R^2^ statistic. Multicollinearity among covariates was evaluated using variance inflation factors. Missing data were minimal and were handled using complete-case analysis.

## 3. Results

### 3.1. Baseline Characteristics of the Study Population

A total of 1226 patients who met the inclusion and exclusion criteria were included in the study. Baseline demographic, clinical, and procedural characteristics are summarized in Table 1. The mean age of the cohort was 62.1 ± 16.8 years, and 49.8% were female. Chronic comorbidities were present in 46.3% of patients. Periampullary duodenal diverticulum was identified in 12.3% of cases. Precut sphincterotomy was performed in 25% of patients, whereas TPS was used in 4.7%.

PEP occurred in 17.3% of patients, hyperamylasemia in 28.5%, bleeding in 2.9%, and perforation in 0.9%. The mean post-procedure hospital stay was 5.8 ± 4.1 days. Laboratory measurements at 24 h post-ERCP showed mean serum levels of amylase (268.94 ± 200.38 U/L), lipase (265.42 ± 190.85 U/L), and C-Reactive Protein (CRP) (53.65 ± 45.84 mg/L).

### 3.2. Impact of Periampullary Diverticulum on ERCP-Related Complications

The association between the presence of periampullary duodenal diverticulum and ERCP-related complications is shown in Table 2. No statistically significant differences were found in PEP (15.2% vs. 17.6%; *p* = 0.49), hyperamylasemia (25.2% vs. 29.0%; *p* = 0.33), bleeding (4.6% vs. 2.6%; *p* = 0.19), or perforation (2.0% vs. 0.7%; *p* = 0.14). Thus, the presence of a diverticulum was not associated with increased complication rates.

### 3.3. Association of Needle–Knife Precut Sphincterotomy with Post-ERCP Complications

As shown in Table 3, precut sphincterotomy was associated with a significantly higher incidence of post-ERCP pancreatitis, with PEP occurring in 30.3% of patients in the precut group compared with 12.9% in the non-precut group, and this difference was statistically significant (*p* < 0.001). This finding indicates a clear association between the use of precut sphincterotomy and the development of PEP in this cohort. In addition to pancreatitis, bleeding was also observed more frequently among patients who underwent precut sphincterotomy, with rates of 4.6% compared with 2.3% in those without precut, representing a statistically significant difference (*p* = 0.04).

In contrast, no statistically significant differences were identified between the precut and non-precut groups with respect to hyperamylasemia or perforation, suggesting that the increased risk associated with precut sphincterotomy in this cohort was primarily driven by pancreatitis and bleeding rather than other ERCP-related adverse events.

### 3.4. Association of TPS with Post-ERCP Complications

The relationship between transpancreatic septotomy and ERCP-related outcomes is presented in Table 4. TPS was strongly associated with a higher incidence of post-ERCP pancreatitis, with PEP occurring in 56.9% of patients in the TPS group compared with 15.3% in the non-TPS group, and this difference was statistically significant (*p* < 0.001). This finding demonstrates a markedly increased risk of pancreatitis among patients requiring TPS in this cohort.

In contrast, no statistically significant differences were observed between the TPS and non-TPS groups with respect to hyperamylasemia, bleeding, or perforation, with *p* values of 0.64, 0.78, and 0.68, respectively. Notably, the overall incidence of perforation remained very low (<1%) in both groups, which inherently limited the statistical power to detect meaningful differences for this rare adverse event.

### 3.5. Effect of Chronic Comorbidities on ERCP-Related Adverse Events

As shown in Table 5, comorbidities were not significantly associated with PEP (15.3% vs. 19%; *p* = 0.09). Hyperamylasemia was significantly lower in patients with comorbidities (25.2% vs. 31.5%; *p* = 0.02). Bleeding was more common in patients with comorbidities (4.2% vs. 1.7%; *p* = 0.007).

### 3.6. Outcomes According to Pancreatic Duct Stent Placement

No significant differences were observed between patients with and without pancreatic duct stents regarding PEP hyperamylasemia perforation or bleeding because all comparisons yielded *p* values above 0.05. Although the proportion of PEP was numerically higher in the stent group the small sample size limited the statistical power to detect a meaningful difference (Table 6).

### 3.7. Independent Predictors of PEP

**Figure 3:**
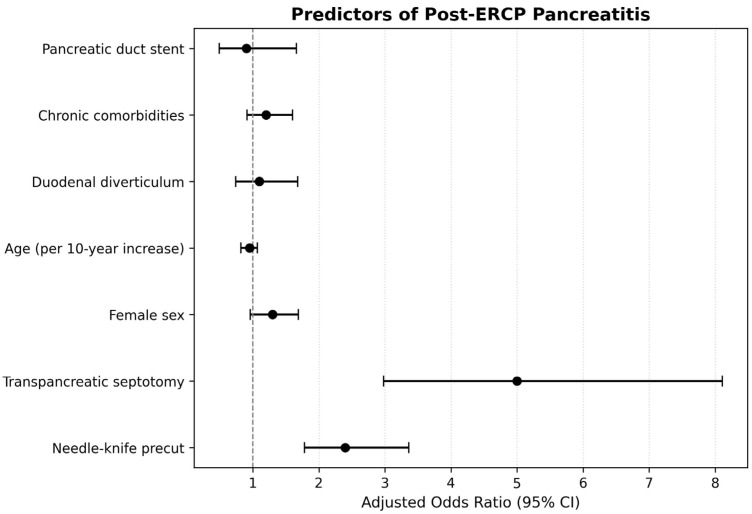
Forest plot illustrating aORs with corresponding 95% CIs for independent predictors of PEP. TPS demonstrated the strongest association with PEP (aOR 4.92, 95% CI 2.98–8.11). Needle–knife precut sphincterotomy was also independently associated with an increased risk of PEP (aOR 2.45, 95% CI 1.78–3.36). Female sex, age, duodenal diverticulum, chronic comorbidities, and pancreatic duct stenting were not significantly associated with PEP, as their CIs crossed the null value of 1.00. The vertical reference line represents an odds ratio of 1.00, indicating no effect. Points represent adjusted odds ratios and horizontal lines represent 95% CIs.

A multivariable logistic regression model (Table 7) identified needle–knife precut sphincterotomy (aOR 2.45; 95% CI 1.78 to 3.36; *p* < 0.001) and TPS (aOR 4.92; 95% CI 2.98 to 8.11; *p* < 0.001) as independent predictors of PEP. TPS demonstrated the highest adjusted effect size and therefore represented the strongest independent risk factor in the model. Female sex showed a nonsignificant trend toward increased risk (aOR 1.28; *p* = 0.08) and age duodenal diverticulum chronic comorbidities and pancreatic duct stenting were not associated with PEP because their CIs included the reference value of 1.00. These associations are illustrated in Figure 3 which displays the aORs as points and the corresponding CIs as horizontal lines and visually highlights the markedly elevated risk associated with TPS and needle–knife precut sphincterotomy. Model calibration was adequate with no evidence of multicollinearity and the model demonstrated moderate explanatory power with a Nagelkerke R squared of 0.19.

## 4. Discussion

This large retrospective study evaluated the relationship between duodenal diverticulum, advanced cannulation techniques (precut and TPS), pancreatic stent placement, comorbidities, and post-ERCP complications in a broad patient population. Our findings provide significant insights into risk factors associated with post-ERCP complications.

The presence of a duodenal diverticulum was not significantly associated with PEP, perforation, or bleeding. This contrasts with some studies suggesting that periampullary diverticula complicate cannulation and increase complication rates (3). Earlier meta-analyses reported no significant increase in PEP, bleeding, or perforation associated with periampullary diverticula [17,18]. Jayaraj et al. further demonstrated that PAD does not significantly affect PEP or major adverse events, despite lower cannulation success [19]. More recent evidence from Xie et al. suggests PAD may increase PEP and bleeding in specific anatomical subtypes such as intradiverticular papilla (IDP). This discrepancy highlights the heterogeneous effect of PAD on ERCP outcomes and underscores operator experience and anatomical variability [4].

The increased incidence of PEP observed with transpancreatic septotomy and needle–knife precut sphincterotomy in our cohort should be interpreted within the context of procedural complexity and difficult biliary cannulation rather than as an inherent risk of these techniques alone. Advanced cannulation methods are typically reserved for cases in which standard biliary access fails, often reflecting prolonged cannulation attempts, repeated pancreatic duct instrumentation, and challenging anatomical conditions, all of which are well-established contributors to PEP risk. Importantly, prior randomized and meta-analytic evidence suggests that the timing and appropriate use of precut techniques may mitigate, rather than exacerbate, procedure-related complications when applied by experienced endoscopists. In a prospective randomized study, Cennamo et al. demonstrated that early implementation of needle–knife precut sphincterotomy did not increase overall complication rates compared with delayed precutting after prolonged standard cannulation attempts, underscoring the role of procedural context rather than technique selection alone [20]. Similarly, a comprehensive meta-analysis of randomized controlled trials by Tang et al. reported that early precut sphincterotomy in patients with difficult biliary access was not associated with an increased risk of PEP and, in fact, was associated with a reduced incidence of PEP compared with persistent cannulation attempts. These findings reinforce the concept that prolonged and repeated cannulation efforts may pose a greater risk than timely transition to advanced access strategies [21].

From a broader pathophysiological perspective, PEP is recognized as a multifactorial complication influenced by both patient-related and procedure-related factors. As reviewed by Tryliskyy et al., mechanical trauma to the papilla, hydrostatic injury from contrast injection, and inflammatory cascades triggered by pancreatic duct manipulation collectively contribute to PEP development. Within this framework, advanced cannulation techniques may serve as markers of increased procedural stress rather than independent causal factors [22]. Consequently, our findings support a clinical approach that emphasizes early risk stratification, judicious selection of advanced cannulation techniques, and the proactive use of prophylactic measures such as pancreatic duct stenting, rectal nonsteroidal anti-inflammatory drugs, and optimized periprocedural hydration in high-risk cases. Together, these strategies may help balance procedural success with complication avoidance in patients requiring advanced biliary access.

Regarding cannulation techniques, both precut and TPS were significantly associated with increased PEP risk. Barakat et al. demonstrated that TPS is safe and effective when performed by experienced endoscopists [7]. In a comparative study evaluating needle–knife fistulotomy, needle–knife papillotomy (NKP), and TPS, PEP rates were 2.6%, 21%, and 22.4%, respectively (*p* = 0.001) [23]. Kylänpää et al. also reported no significant difference in PEP rates between TPBS and DGW, while TPBS achieved higher cannulation success. Multivariable analysis in our study confirmed that both precut and TPS independently increased PEP risk [24].

Recent systematic reviews and meta-analyses reinforce this interpretation. A comprehensive Beran et al. meta-analysis identified precut sphincterotomy, difficult cannulation, pancreatic duct cannulation, and acinarization as major predictors of PEP [12]. Similarly, the foundational meta-analysis by Ding et al. reported significantly elevated risk with difficult cannulation (OR 3.49), precut sphincterotomy (OR 2.25), and pancreatic duct injection (OR 1.58) [11]. These findings align closely with our results and indicate that advanced cannulation techniques largely reflect procedural complexity rather than intrinsic technique-related risk.

TPS, while a valuable alternative in cases of failed standard cannulation, carries unique disadvantages, particularly the risk of pancreatic duct trauma provoking edema and inflammation. Literature consistently reports higher PEP incidence in TPS patients [25]. Prolonged hospital stays observed in our TPS group also mirror prior data [26,27]. However, recent studies including Vezakis et al. show that needle–knife sphincterotomy does not independently increase PEP risk after adjusting for procedural difficulty but is associated with higher perforation rates [28]. Similarly, Lyu et al. demonstrated comparable PEP and perforation rates between TPS and NKP, with superior cannulation success in TPS [29]. Notably, Su et al. found that TPS significantly improves cannulation rates even in low-volume centers without increasing PEP, highlighting the importance of operator familiarity with the technique [13]. Nevertheless, in the absence of objective and standardized measures of cannulation difficulty in the present study, the observed association between TPS and post-ERCP pancreatitis should be interpreted with caution. The markedly elevated PEP rate in the TPS group cannot be definitively attributed to the intrinsic risk of the technique itself and likely reflects extreme procedural complexity and indication bias rather than a direct causal effect. Together, these findings suggest the elevated PEP rate seen with TPS in our cohort likely reflects extreme procedural difficulty rather than inherent technique-related risk.

In our study, TPS was not significantly associated with hyperamylasemia, bleeding, CRP, or lipase levels, supporting multifactorial mechanisms underlying PEP. No significant differences were found between TPS and non-TPS groups regarding age, sex, comorbidities, or cholecystectomy history, implying technical rather than patient factors drive TPS utilization [16,30]. Dumonceau et al. similarly emphasized procedural technique over patient characteristics in determining ERCP risk [31].

Prophylactic pancreatic stent placement did not demonstrate a protective effect in our cohort, contrasting with earlier evidence [8,32]. These results closely mirror the findings of Sperna-Weiland et al., whose individual patient data meta-analysis showed that pancreatic stents do not reduce PEP risk after adjustment (RR 1.25), whereas rectal NSAIDs and aggressive hydration do [9]. A clinical cohort study by Ghalehnoei et al. also found no difference in PEP rates between patients receiving rectal indomethacin alone and those receiving prophylactic pancreatic stents. These observations suggest that stent efficacy may be overestimated in unadjusted analyses and that optimal prophylaxis should prioritize NSAIDs and hydration [24].

Chronic comorbidities were associated with a higher risk of post-ERCP bleeding in our cohort, emphasizing the importance of individualized pre-procedural assessment, particularly in patients with systemic disease. In contrast, hyperamylasemia was more frequently observed in patients without comorbidities, suggesting that isolated pancreatic enzyme elevation may reflect procedure-related pancreatic irritation rather than patient-related risk factors. Consistent with this observation, prior studies have demonstrated that patient-related characteristics such as age, sex, and chronic comorbidities show inconsistent associations with PEP across heterogeneous clinical populations. In a cohort study comparing prophylactic pancreatic stenting and rectal indomethacin, Ghalehnoei et al. found that most patient-related factors were not independently associated with PEP, whereas deep pancreatic duct cannulation was the primary procedural risk factor [33]. Similarly, a large national database analysis by Gordon et al. demonstrated that comorbid conditions predominantly influenced overall clinical outcomes, including mortality and length of hospital stay, rather than pancreatitis-specific risk. Together, these findings suggest that systemic comorbidities may play a more prominent role in hemorrhagic and global post-ERCP outcomes, while procedure-related factors remain the principal determinants of pancreatic injury [34].

This study has several limitations that should be considered when interpreting the findings. First, the retrospective single-center design introduces inherent risks of selection bias, unmeasured confounding, and operator-dependent variability. Although all ERCP procedures were performed by experienced endoscopists, differences in individual technique, risk tolerance, and decision-making may have influenced the use of advanced cannulation strategies such as precut and TPS. In addition, objective and standardized measures of cannulation difficulty, including cannulation time, number of cannulation attempts, and unintended pancreatic duct cannulations, were not consistently available due to the retrospective nature of the study and therefore could not be systematically analyzed. Second, the study did not include detailed stratification of periampullary diverticula based on size, subtype, or papillary orientation, factors known to influence cannulation difficulty and complication risk. Third, the relatively small number of patients undergoing TPS (n = 58) may have limited the precision of subgroup analyses despite demonstrating statistically significant associations. Fourth, although pancreatic duct stenting was evaluated, information about stent caliber, length, placement technique, and timing of migration/removal was not available, preventing assessment of technical or device-related contributors to outcomes. Furthermore, detailed data regarding the systematic use of established prophylactic measures for post-ERCP pancreatitis, including rectal nonsteroidal anti-inflammatory drugs and aggressive intravenous hydration, were not uniformly available in the retrospective dataset, limiting assessment of their potential modifying effect on PEP risk. Finally, rare complications such as perforation occurred infrequently (<1%), which inherently limits the ability to detect meaningful between-group differences or evaluate risk factors through multivariable modeling. Despite these limitations, the large sample size and comprehensive procedural documentation strengthen the validity of the results and provide clinically meaningful insights into factors affecting PEP and other ERCP-related complications.

Future research should prioritize prospective, multicenter cohort studies and randomized clinical trials to more accurately assess the causal relationship between advanced cannulation techniques and post-ERCP complications. Detailed anatomical characterization of periampullary diverticula using endoscopic and radiologic criteria may help clarify their true impact on cannulation success and adverse event risk. Given the heterogeneous safety profiles of precut and TPS, future studies should explore standardized cannulation algorithms that incorporate real-time risk stratification tools and operator experience metrics. Furthermore, individual patient data meta-analyses suggest that prophylactic pancreatic stenting may offer limited benefit when adjusted for baseline risk; therefore, future investigations should focus on optimizing pharmacologic prophylaxis, evaluating combinations of rectal NSAIDs, aggressive hydration, and novel anti-inflammatory strategies. Development of validated prediction models that integrate patient-related, anatomical, and procedure-specific variables could assist clinicians in identifying high-risk individuals and selecting the safest and most effective cannulation technique. Ultimately, expanding the evidence base surrounding TPS, particularly through large-scale randomized trials, will be essential to defining its role within modern ERCP practice and improving patient safety.

## 5. Conclusions

In this large single-center cohort, procedural factors such as TPS and needle–knife precut sphincterotomy were identified as the strongest independent predictors of PEP, and TPS showed the highest risk. In contrast, periampullary duodenal diverticulum and pancreatic duct stenting were not associated with an increased risk of PEP. Comorbidities were mainly related to a higher likelihood of bleeding rather than PEP.

These findings indicate that the development of PEP is influenced more by the difficulty of cannulation and the need for advanced access techniques than by patient related or anatomical characteristics. Careful patient selection, consistent use of standardized cannulation strategies and the application of evidence-based prophylactic measures are essential to reducing the incidence of ERCP related adverse events.

Prospective multicenter studies and randomized clinical trials are needed to confirm these results and to further clarify the optimal role of TPS and precut sphincterotomy in modern ERCP practice.

## Figures and Tables

**Table 1:** Demographic, clinical, and procedural characteristics of the study population (n = 1226).

Variable	Mean ± SD/n (%)	Range
Age (years)	62.1 ± 16.8	18–100
Post-ERCP hospital stay (days)	5.8 ± 4.1	1–80
Male	616 (50.2%)	
Female	610 (49.8%)	
Comorbidities present	568 (46.3%)	
Comorbidities absent	658 (53.7%)	
Duodenal diverticulum present	151 (12.3%)	
Duodenal diverticulum absent	1075 (87.7%)	
Needle–knife precut	307 (25.0%)	
Transpancreatic septotomy (TPS)	58 (4.7%)	
Post-ERCP PEP	212 (17.3%)	
– Mild	140 (66%)	
– Moderate	43 (20%)	
– Severe	29 (13%)	
Hyperamylasemia	350 (28.5%)	
Bleeding	35 (2.9%)	
Perforation	11 (0.9%)	
Amylase at 24 h (U/L)	268.94 ± 200.38	
Lipase at 24 h (U/L)	265.42 ± 190.85	
CRP at 24 h (mg/L)	53.65 ± 45.84	

**Table 2:** Association between periampullary duodenal diverticulum and ERCP-related complications.

Complication	Diverticulum Present n (%)	Diverticulum Absent n (%)	*p*-Value
PEP	23 (15.2%)	189 (17.6%)	0.49
Hyperamylasemia	38 (25.2%)	321 (29.0%)	0.33
Perforation	3 (2.0%)	8 (0.7%)	0.14
Bleeding	7 (4.6%)	28 (2.6%)	0.19

**Table 3:** Association between needle–knife precut and post-ERCP complications.

Complication	Pre-Cut Performed n (%)	Pre-Cut Not Performed n (%)	*p*-Value
PEP	93 (30.3%)	119 (12.9%)	<0.001 *
Hyperamylasemia	76 (24.8%)	274 (29.8%)	0.09
Perforation	3 (1.0%)	8 (0.9%)	0.99
Bleeding	14 (4.6%)	21 (2.3%)	0.04 *

* Statistically significant (*p* < 0.05).

**Table 4:** Association between TPS and complications.

Complication	TPS Present n (%)	TPS Absent n (%)	*p*-Value
PEP	33 (56.9%)	179 (15.3%)	<0.001 *
Hyperamylasemia	15 (25.9%)	335 (28.7%)	0.64
Perforation	0 (0.0%)	11 (0.9%)	0.68
Bleeding	2 (3.4%)	33 (2.8%)	0.78

* Statistically significant (*p* < 0.05).

**Table 5:** Association between chronic comorbidities and post-ERCP complications.

Complication	Comorbidities Present n (%)	Comorbidities Absent n (%)	*p*-Value
PEP	87 (15.3%)	125 (19.0%)	0.09
Hyperamylasemia	143 (25.2%)	207 (31.5%)	0.02 *
Perforation	7 (1.2%)	4 (0.6%)	0.36
Bleeding	24 (4.2%)	11 (1.7%)	0.007 *

* Statistically significant (*p* < 0.05).

**Table 6:** Association between pancreatic duct stenting and post-ERCP complications.

Complication	Stent Present n (%)	Stent Absent n (%)	*p*-Value
PEP	5 (31.3%)	207 (17.1%)	0.17
Hyperamylasemia	7 (43.8%)	343 (28.3%)	0.26
Perforation	0 (0.0%)	11 (0.9%)	0.86
Bleeding	0 (0.0%)	35 (2.9%)	0.99

**Table 7:** Multivariable logistic regression for predictors of PEP.

Predictor	Adjusted OR (aOR)	95% CI	*p*-Value
Needle–knife precut	2.45	1.78–3.36	<0.001 *
Transpancreatic septotomy (TPS)	4.92	2.98–8.11	<0.001 *
Female sex	1.28	0.96–1.69	0.08
Age (per 10-year increase)	0.94	0.82–1.07	0.35
Duodenal diverticulum	1.12	0.74–1.68	0.59
Chronic comorbidities	1.21	0.91–1.60	0.17
Pancreatic duct stent	0.91	0.49–1.66	0.76

* Statistically significant (*p* < 0.05).

## Data Availability

The datasets generated and/or analyzed during the current study are available from the corresponding author on reasonable request.

## Abbreviations

The following abbreviations are used in this manuscript:

CIs	Confidence intervals
ERCP	Endoscopic Retrograde Cholangiopancreatography
PEP	Post-ERCP Pancreatitis
NSAIDs	Nonsteroidal Anti-Inflammatory Drugs
CRP	C-Reactive Protein
TPS	Transpancreatic Septotomy (Goff technique)
NKP	Needle–Knife Papillotomy
COPD	Chronic Obstructive Pulmonary Disease
aORs	Adjusted Odds Ratios
